# TSPO Ligands PK11195 and Midazolam Reduce NLRP3 Inflammasome Activation and Proinflammatory Cytokine Release in BV-2 Cells

**DOI:** 10.3389/fncel.2020.544431

**Published:** 2020-12-10

**Authors:** Hao Feng, Yongxin Liu, Rui Zhang, Yingxia Liang, Lina Sun, Nannan Lan, Baoyu Ma

**Affiliations:** Shandong Provincial Medicine and Health Key Laboratory of Clinical Anesthesia, School of Anesthesiology, Weifang Medical University, Weifang, China

**Keywords:** TSPO, NLRP3 inflammasome, PK11195, midazolam, neurodegenerative diseases, neuroinflammation

## Abstract

Neuroinflammation related to microglial activation plays an important role in neurodegenerative diseases. Translocator protein 18 kDa (TSPO), a biomarker of reactive gliosis, its ligands can reduce neuroinflammation and can be used to treat neurodegenerative diseases. Therefore, we explored whether TSPO ligands exert an anti-inflammatory effect by affecting the nucleotide-binding domain-like receptor protein 3 (NLRP3) inflammasome, thereby inhibiting the release of inflammatory cytokines in microglial cells. In the present study, BV-2 cells were exposed to lipopolysaccharide (LPS) for 6 h to induce an inflammatory response. We found that the levels of reactive oxygen species (ROS), NLRP3 inflammasome, interleukin-1β (IL-1β), and interleukin-18 (IL-18) were significantly increased. However, pretreatment with TSPO ligands inhibited BV-2 microglial and NLRP3 inflammasome activation and significantly reduced the levels of ROS, IL-1β, and IL-18. Furthermore, a combination of LPS and ATP was used to activate the NLRP3 inflammasome. Both pretreatment and post-treatment with TSPO ligand can downregulate the activation of NLRP3 inflammasome and IL-1β expression. Finally, we found that TSPO was involved in the regulation of NLRP3 inflammasome with TSPO ligands treatment in TSPO knockdown BV2 cells. Collectively, these results indicate that TSPO ligands are promising targets to control microglial reactivity and neuroinflammatory diseases.

## Introduction

The incidence of neurodegenerative diseases increases with age, and the complications of neurodegenerative diseases seriously affect the quality of life and survival rate of the elderly (Karim et al., 2014). The causes of neurodegenerative diseases are unclear, and treatments for neurodegenerative diseases are very limited. Recent research suggests that the development of neuroinflammation is closely related to a variety of neurodegenerative diseases (Ransohoff, 2016). The occurrence and development of neuroinflammation are closely related to the activation of microglia (Subhramanyam et al., 2019). Activated microglia can produce inflammatory cytokines, including tumor necrosis factor-alpha (TNF-α), interleukin-1β (IL-1β), and reactive oxygen species (ROS), which could result in the progression of neurodegenerative diseases (Xu et al., 2016). Also, research has shown that oxidative stress is associated with the pathogenesis of neurodegenerative diseases (Crotty et al., 2017). Mitochondria-derived ROS may activate the nucleotide-binding domain-like receptor protein 3 (NLRP3) inflammasome (Wang et al., 2017). NLRP3 inflammasome is a multi-protein complex that is distributed in the cytosol (Tschopp and Schroder, 2010). The NLRP3 inflammasome consists of NLRP3, an apoptosis-associated speck-like protein containing a caspase recruitment domain (ASC), and cysteinyl aspartate specific proteinase 1 (caspase-1). This inflammasome induces caspase-1 activation, which induces the maturation and secretion of proinflammatory cytokines, including IL-1β and interleukin-18 (IL-18; Li et al., 2017). IL-1β and IL-18 initiate a variety of signaling pathways and drive inflammation, which leads to neuronal damage or death (Yatsiv et al., 2002; Meissner et al., 2010; Wilms et al., 2010). In the central nervous system (CNS), the inappropriate activation of the NLRP3 inflammasome participates in the pathogenesis of both acute and chronic neurodegenerative conditions (Chen et al., 2015; Ito et al., 2015). Translocator protein 18 kDa (TSPO), which is a new name for a peripheral benzodiazepine receptor, has been studied as a biomarker of reactive gliosis and inflammation associated with a variety of neuropathological conditions (Chen and Guilarte, 2008). TSPO plays an important role in regulating inflammation (McNeela et al., 2018). Under normal physiological conditions, TSPO expression is low in brain glial cells but is significantly increased in brain injury and inflammation, a feature that makes it particularly suitable for assessing active glial cells (Biswas et al., 2018; Werry et al., 2019). Significant increases in TSPO expression were found in many neurodegenerative diseases, such as Alzheimer’s disease (AD; Metaxas et al., 2019), Parkinson’s disease (PD; Gerhard, 2016), and Huntington’s disease (HD; Metaxas et al., 2019). However, the causal relationship between changes in TSPO protein expression and the occurrence of neurodegenerative diseases is still unclear. Recent evidence indicates that TSPO can be used as a biomarker for neuroinflammation in the brain and that TSPO ligands can be targeted to induce therapeutic effects against neurological disease (Kim and Pae, 2016). Also, TSPO ligands show therapeutic effects in terms of neuroprotection and anxiety (Rupprecht et al., 2010). PK11195, a classic TSPO ligand, shows great potential in treating neurodegenerative diseases (Azrad et al., 2019). Midazolam, which is a benzodiazepine, can inhibit the release of neuroinflammatory factors (Tanabe et al., 2011). In this study, we examined the effects of PK11195 and midazolam pretreatment or post-treatment on neuroinflammation and determined that the NLRP3 inflammatory pathway is involved in these effects.

## Materials and Methods

### Cell Culture and Treatment

The BV-2 mouse microglial cell line was purchased from the Cobioer Biological Technology Company (Nanjing, China). BV-2 cells were cultured in 1,640 medium with 10% fetal bovine serum (Gibco, Shanghai, China) and 1% penicillin-streptomycin in an atmosphere with 5% CO_2_ at 37°C. To investigate the anti-inflammatory effects of PK11195 and midazolam pretreatment, BV-2 cells were exposed to 1 μg/ml lipopolysaccharide (LPS; Solarbio, Beijing, China) for 6 h to stimulate the inflammatory response and constitute the LPS group. BV-2 cells incubated with culture medium served as the control group. BV-2 microglial cells were pretreated with 0.5 μM PK11195 (Sigma–Aldrich, St. Louis, MO, USA) or 15 μM midazolam (NHWA, Xuzhou, Jiangsu, China) for 1 h and then exposed to LPS for 6 h. These two groups were used as the PK11195 + LPS group and midazolam + LPS group. For the PK11195 group and midazolam group, BV-2 cells were previously incubated with 0.5 μM PK11195 or 15 μM midazolam for 6 h. We also used a combination of LPS and ATP to stimulate BV2 cells. BV-2 cells were incubated with LPS (1 μg/ml) for 6 h followed by 1 mM ATP (Sigma–Aldrich, St. Louis, MO, USA) treated for 2 h and constitute the LPS + ATP group. BV-2 microglial cells were pretreated with 0.5 μM PK11195 or 15 μM midazolam for 1 h and then exposed to LPS + ATP. These two groups were used as the PK11195 + LPS + ATP group and midazolam + LPS + ATP group. To investigate the anti-inflammatory effects of PK11195 and midazolam post-treatment, the cells were treated with PK11195 (0.5 μM) or midazolam (15 μM) for 2 h, after stimulated with LPS + ATP. These two groups were used as the LPS + ATP + PK11195 group and LPS + ATP + midazolam group.

### Western Blot Analysis

After treatment, the cells were collected and lysed in RIPA buffer (Solarbio, Beijing, China) containing PMSF (Solarbio, Beijing, China) buffer. Protein (30 μg) was extracted and electrophoresed on SDS–PAGE gels and transferred to PVDF membranes (Millipore Corp., Bedford, MA, USA). The membranes were blocked with PBS buffer containing 5% skim milk at room temperature for 2 h and then incubated overnight at 4°C with specific antibodies against NLRP3 (1:1,000; Abcam, Cambridge, UK), caspase-1 (1:1,000; Abcam, Cambridge, UK), ASC (1:100; Santa Cruz Biotechnology, Dallas, TX, USA), IBA-1 (1:1,000; Abcam, Cambridge, UK), caspase-1 p10 (1:1,000; Abcam, Cambridge, UK), IL-1β (1:1,000; Abcam, Cambridge, UK); TSPO (1:1,000; Abcam, Cambridge, UK) or β-actin (1:2,000; Beyotime, Shanghai, China). After washing the membranes with PBS, horseradish peroxidase-conjugated secondary antibody diluted 7,000-fold was incubated with the membrane at room temperature for 1 h. Then, the membranes were observed using a Tanon 5200 multifunctional imaging system. β-actin was used as a loading control.

### Detection of Mitochondrial-Derived DCFH-ROS Levels

The radicals ROS production was measured by DCFH-DA staining. LPS-induced BV-2 cells were pretreated with PK11195 or midazolam. The cells were washed with PBS three times and incubated with 2.5 μM DCFH-DA (Beyotime, Shanghai, China) in the dark at 37°C for 30 min. After washing the cells three times, images were captured using a fluorescence microscope (Olympus, Tokyo, Japan) and analyzed with Image-Pro Plus. The excitation wavelength was 488 nm, and the emission wavelength was 525 nm.

### Enzyme-Linked Immunosorbent Assay (ELISA)

BV-2 cells were exposed to 1 μg/ml LPS for 6 h to stimulate the inflammatory response. For the LPS groups, cells incubated with culture medium served as the control. BV-2 microglial cells in the LPS + PK11195 or LPS + midazolam groups were pretreated with 0.5 μM PK11195 or 15 μM midazolam for 1 h and then exposed to LPS for 6 h. Subsequently, the medium of the different groups was collected. IL-1β (Dakewe, Beijing, China) and IL-18 (Multi Sciences, Hangzhou, Zhejiang, China) that the cells had secreted into the culture supernatant were measured by ELISAs according to the manufacturer’s instructions. The optical density (OD) values at 450 nm were measured using a microplate reader.

### Immunocytochemistry

After treatment, the cells were fixed with 4% paraformaldehyde (PFA) in PBS for 30 min, washed three times with PBS for 5 min each time, and then blocked in 5% goat blocking serum (Solarbio, Beijing, China) for 30 min at room temperature. The cells were then incubated overnight with anti-NLRP3 (1:100; Abcam, Cambridge, UK), anti-ASC (1:100; Santa Cruz Biotechnology, Dallas, TX, USA), anti-caspase-1 (1:150; Abcam, Cambridge, UK), and anti-IBA-1 antibodies (1:100; Abcam, Cambridge, UK) at 4°C. After washing three times with PBS, the cells were incubated with goat anti-rabbit IgG (1:500; Multi Sciences, Hangzhou, Zhejiang, China) or goat anti-mouse IgG (1:500; Multi Sciences, Hangzhou, Zhejiang, China) secondary antibody for 2 h in the dark at 37°C. Finally, fluorescence images were acquired with a fluorescence microscope (Olympus, Tokyo, Japan), and the analysis of the fluorescence images was performed by ImageJ.

### RNA Isolation and Real-Time PCR Assays

Total RNA was extracted with TRIzol reagent (Thermo Fisher Scientific, Shanghai, China). Reverse transcription was performed with a ReverTra Ace qPCR RT kit (Toyobo, Osaka, Japan) according to the manufacturer’s instructions. For real-time PCR analysis, the resultant cDNA products were amplified using a 2× ChamQ SYBR qPCR Master Mix in triplicate. β-Actin was used for standardization. The forward and reverse primer sequences are shown in Table 1. RT-PCR was performed for 40 cycles at 95°C for 15 s and 60°C for 1 min after an initial 15 min incubation at 95°C.

**Table 1:** Primers used in the study.

Gene	Forward primers	Reverse primers
NLRP3	CCTGGGGGACTTTGGAATCAG	GATCCTGACAACACGCGGA
Caspase-1	TTGAGGGTCCCAGTCAGTCC	CCCCAGGCAAGCCAAATC
IL-1β	GCCCATCCTCTGTGACTCAT	AGGCCACAGGTATTTTGTCG
IL-18	GCCTGTGTTCGAGGATATGACT	CCTTCACAGAGAGGGTCACAG
TSPO	GCTGTGGATCTTTCCAGAACA	ATGCCAAGAGGGTTTCTGC
β-actin	CCAGTTGGTAACAATGCCATGT	GGCTGTATTCCCCTCCATCG

### siRNA Preparation and Transfection

For siRNA experiments, BV-2 cells (6 × 10^5^/well) were seeded onto six-well plates. After 24 h, cells were transfected with siRNA (100 nM; Genechem, Shanghai, China). The sequence of siRNA against TSPO was as follows:

mTSPO-F: 5′-CCGUGCUCAACUACUAUGUAUTT-3′.

mTSPO-R: 5′-AUACAUAGUAGUUGAGCACGGTT-3′.

The siRNAs were transfected using Lipofectamine 3000 (Invitrogen, Carlsbad, CA, USA) according to the manufacturer’s instructions. After 6 h, the medium was changed and the cells were incubated with fresh medium for 24 h. The levels of TSPO in transfected BV-2 cells were analyzed by western blot and real-time PCR.

### Statistical Analysis

All values are expressed as the means ± SEM. The data were analyzed with one-way ANOVA followed by Tukey’s *post hoc* test for significance *via* SPSS 20.0. The results were considered significant when *P* < 0.05.

## Results

### PK11195 and Midazolam Reduce ROS Levels and Inhibit NLRP3 Inflammasome Activation

**Figure 1:**
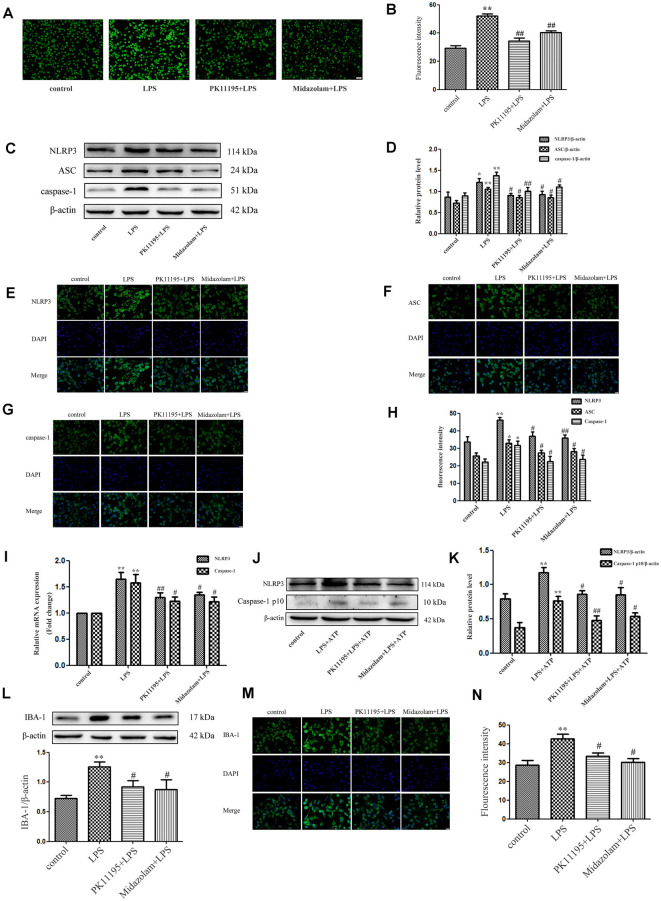
PK11195 and midazolam reduced the reactive oxygen species (ROS) levels and inhibit the activation of NLRP3 inflammasome in the lipopolysaccharide (LPS)-stimulated or LPS + ATP induced BV-2 cells. Cells were pretreated with PK11195 (0.5 μM) or midazolam (15 μM) for 1 h and then with LPS (1 μg/ml) for another 6 h. **(A)** Representative images showing the ROS expression with DCFH-DA in the LPS-stimulated BV-2 cells. Scale bar, 40 μm. **(B)** Quantification of the fluorescence intensity of the ROS using Image-Pro Plus (*n* = 5). **(C)** Representative images of NLRP3, ASC, caspase-1, and β-actin expression in LPS-stimulated BV-2 cells by western blot. **(D)** Comparison of NLRP3, ASC, and caspase-1 expression in the LPS-stimulated BV-2 cells in each group based on western blot analysis (*n* = 5). Representative images showing NLRP3 **(E)**, ASC **(F)**, and caspase-1 **(G)** expression with immunocytochemistry in the LPS-stimulated BV-2 cells. Scale bar, 20 μm. **(H)** Quantification of the fluorescence intensity of NLRP3 using Image-Pro Plus (*n* = 5). **(I)** Comparison of NLRP3 and caspase-1 expression in the LPS-stimulated BV-2 cells by group using real-time PCR (*n* = 5). Cells were pretreated with PK11195 (0.5 μM) or midazolam (15 μM) for 1 h, then exposed to LPS (1 μg/ml) for 6 h, followed by ATP (1 mM) incubation for another 2 h. *P < 0.05, **P < 0.01, compared with the control group; ^#^*P* < 0.05, ^##^*P* < 0.01, compared with the LPS group. **(J)** Representative images of NLRP3 and caspase-1 p10 expression in LPS + ATP induced BV-2 cells by western blot. **(K)** Comparison of NLRP3 and caspase-1 p10 expression in the LPS + ATP induced BV-2 cells in each group based on western blot analysis (*n* = 5). *P < 0.05, **P < 0.01, compared with the control group; ^#^*P* < 0.05, ^##^*P* < 0.01, compared with the LPS + ATP group. **(L)** Comparison of IBA-1 expression in the BV-2 cells by a group based on western blot analysis (*n* = 5). **(M)** Representative immunocytochemistry of IBA-1 expression in the BV-2 cells. Scale bar, 20 μm. **(N)** Quantification of the fluorescence intensity of IBA-1 using Image-Pro Plus (*n* = 5). **P < 0.01, compared with the control group; ^#^*P* < 0.05, compared with the LPS group.

We first investigated whether pretreatment with the TSPO ligands PK11195 and midazolam mediated the ROS production in BV-2 cells. The results showed that the levels of ROS were significantly increased in the cells stimulated with LPS (1 μg/ml) for 6 h. However, PK11195 or midazolam pretreatment significantly inhibited the expression of ROS stimulated by LPS. The results indicated that PK11195 and midazolam inhibit the production of ROS in microglial cells (Figures 1A,B). To further investigate whether the NLRP3 inflammasome involved in the effect regulated by PK11195 and midazolam in microglia, the levels of NLRP3, ASC, and caspase-1 were measured by western blot, RT-PCR, and immunocytochemistry. The results showed that LPS stimulation observably increased the levels of NLRP3, ASC, and caspase-1, whereas PK11195 or midazolam pretreatment significantly inhibited the protein expression of NLRP3, ASC, and caspase-1 and the mRNA expression of NLRP3 and caspase-1 (Figures 1C–I). Activation of NLRP3 inflammasome requires priming and activating signals. Many studies show that LPS and ATP are priming and activating stimuli, respectively, and are used in combination to activate NLRP3 inflammasome (Song et al., 2017; Heneka et al., 2018; Kelley et al., 2019). Next, we used LPS + ATP combination to induce NLRP3 inflammasome and further verify NLRP3 inflammasome was involved in the anti-inflammatory effects of PK11195 and midazolam. We found that NLRP3 and Caspase-1 p10 were dramatically up-regulated in LPS + ATP, but were significantly decreased with PK11195 or midazolam intervention (Figures 1J,K). These results further verified that pretreatment with PK11195 or midazolam inhibited the activation of the NLRP3 inflammasome in microglia. To examine whether pretreatment with PK11195 or midazolam inhibited microglial activation, the expression of IBA-1 was assayed using western blot and immunocytochemistry analyses (Figures 1L–N). We found that the levels of IBA-1 were upregulated by LPS stimulation, but effectively inhibited by pretreatment with PK11195 or midazolam. Meanwhile, the effects of PK11195 or midazolam alone on NLRP3 inflammasomes and IBA-1 in the basal activity of BV-2 cells were assayed using western blot analysis. We found that the levels of NLRP3 inflammasomes and IBA-1 were no significant changes in the basal state of BV-2 cells treated with PK11195 or midazolam alone (Supplementary Figure 1).

### PK11195 and Midazolam Reduced NLRP3-Mediated IL-1β and IL-18 Secretion

**Figure 2:**
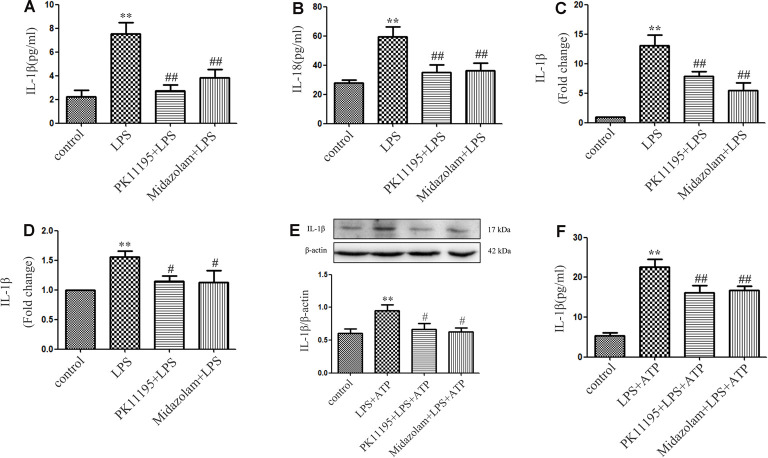
PK11195 or midazolam inhibited the expression of IL-1β and IL-18 in the LPS-stimulated or LPS + ATP induced BV-2 cells. Cells were pretreated with PK11195 (0.5 μM) or midazolam (15 μM) for 1 h and with LPS (1 μg/ml) for another 6 h. Comparison of IL-1β **(A)** and IL-18 **(B)** expression in the LPS-stimulated BV-2 cells by a group based on enzyme-linked immunosorbent assay (ELISA; *n* = 5). Comparison of IL-1β **(C)** and IL-18 **(D)** expression in the LPS-stimulated BV-2 cells by group using real-time PCR. Cells were pretreated with PK11195 (0.5 μM) or midazolam (15 μM) for 1 h, then exposed to LPS (1 μg/ml) for 6 h, followed by ATP (1 mM) incubation for another 2 h. **P < 0.01, compared with the control group; ^#^*P* < 0.05, ^##^*P* < 0.01, compared with the LPS group. **(E)** Comparison of IL-1β expression in the LPS + ATP induced BV-2 cells by a group based on western blot analysis (*n* = 5). **(F)** Comparison of IL-1β release in the medium of LPS + ATP induced BV-2 cells by a group based on ELISAs (*n* = 5). **P < 0.01, compared with the control group; ^#^*P* < 0.05, ^##^*P* < 0.01, compared with the LPS + ATP group.

Moreover, we tested whether PK11195 or midazolam can inhibit NLRP3-mediated IL-1β and IL-18 secretion in microglial cells. BV-2 microglial cells were pretreated with PK11195 or midazolam for 1 h and then exposed to LPS for 6 h. The results showed that the levels of IL-1β and IL-18 were significantly increased in the LPS-treated BV-2 microglial cells in ELISAs and the RT-PCR analysis. However, pretreatment with PK11195 or midazolam effectively inhibited the expression of IL-1β and IL-18 (Figures 2A–D). To further investigate the inhibitor effect of PK11195 or midazolam on IL-1β related to NLRP3 inflammasome, BV2 cells were stimulated by LPS + ATP and treated with PK11195 or midazolam. Consistent with the literature, the levels of NLRP3 and Caspase-1 p10 were robustly upregulated by LPS + ATP, indicating the activation of the NLRP3 inflammasome (Li et al., 2018). As expected, pretreatment with PK11195 or midazolam significantly reduced IL-1β levels in cell lysates and medium (Figures 2E,F). These results suggest that pretreatment with PK11195 and midazolam inhibited the secretion of the inflammatory cytokines measured associated with NLRP3.

Also, we observed the inhibitory effect of TSPO ligand on NLRP3 inflammasome using post-treatment of PK11195 and midazolam. The cells were firstly incubated with LPS for 6 h and then treated with PK11195 or midazolam for extra 2 h following ATP treatment for 2 h. The cell lysates were collected and applied to western blotting, and the cytokine in the medium was assayed by ELISAs. Consistent with our previous study, the levels of NLRP3 and Caspase-1 p10 were significantly increased in LPS + ATP-stimulated BV-2 cells. Post-treatment of PK11195 or midazolam significantly reversed the upregulation of the NLRP3 and caspase-1 p10 (Supplementary Figures 2A,B) induced by LPS + ATP. Furthermore, posttreatment of PK11195 and midazolam significantly down-regulated NLRP3 inflammasome-derived IL-1β release (Supplementary Figures 2C,D). These results indicate that post-treatment with PK11195 and midazolam inhibited the activation of NLRP3 inflammasome and downregulated IL-1β release.

### Effects of PK11195 and Midazolam on Activation of NLRP3 Inflammasome in TSPO Knockdown Microglia

**Figure 3:**
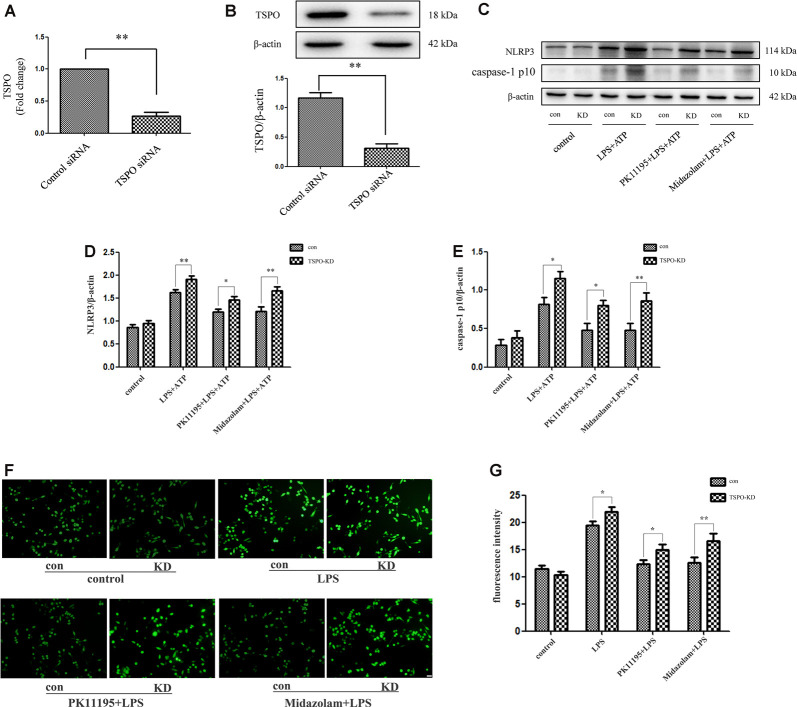
TPSO ligands PK11195 or midazolam downregulated the activation of NLRP3 inflammasome in BV2 cells by a TSPO-dependent mechanism. TSPO knockdown BV2 cells were established using siRNA transfection. **(A,B)** Comparison of TSPO mRNA and protein expression in TSPO knockdown cells and the control siRNA cells (*n* = 5). Both TSPO knockdown and the control siRNA cells were pretreated with 0.5 μM PK11195 or 15 μM midazolam for 1 h and then exposed to LPS (1 μg/ml) for 6 h, followed by ATP (1 mM) incubation for another 2 h. **(C)** Representative images of NLRP3, caspase-1 p10, and β-actin expression on control siRNA and TSPO siRNA BV-2 cells by western blot. **(D,E)** Comparison of the levels of NLRP3 **(D)** and caspase-1 p10 **(E)** in the TSPO knockdown and control siRNA BV-2 cells by a group based on western blot analysis (*n* = 5). **(F)** Representative images showing the ROS expression with DCFH-DA in control siRNA and TSPO siRNA BV-2 cells. Scale bar, 40 μm. **(G)** Quantification of the fluorescence intensity of the ROS using Image-Pro Plus (*n* = 5). *P < 0.05, **P < 0.01.

To further examine whether TSPO was involved in the regulation of NLRP3 inflammasome with TSPO ligands treatment, we established TSPO knockdown BV2 cells using siRNA transfection. Analysis of mRNA and protein expression revealed a significant reduction of TSPO in transduced cells compared with the control cells (Figures 3A,B). Both TSPO knockdown and the control cells were pretreated with 0.5 μM PK11195 or 15 μM midazolam for 1 h and then exposed to LPS (1 μg/ml) for 6 h, followed by ATP (1 mM) incubation for another 2 h. The cell lysates were collected and applied to western blot analysis. The results showed that the levels of ROS, NLRP3, and cleaved-caspase-1 stimulated by LPS + ATP in TSPO knockdown cells were significantly higher than those in the control group. Moreover, pretreatment with PK11195 or midazolam reduced the production of ROS and decreased the NLRP3 and cleaved-caspase-1 expression in the control cells, although TSPO knockdown cells were affected too. However, the levels of the two groups were significantly different (Figures 3C–G). These results indicated that TSPO was involved in the regulation of ROS and NLRP3 inflammasome and in the modulation of inflammation with TSPO ligands treatment.

## Discussion

To study the effects of TSPO ligands on neurodegenerative diseases related to neuroinflammation, we performed experiments on the effects of TSPO ligands treatment on inflammatory factors and concluded that treatment with TSPO ligands can inhibit the progression of neuroinflammation. Our current study shows that treatment of TSPO ligands PK11195 and midazolam can inhibit the activation of BV-2 microglia and the production of ROS, thereby also inhibiting the expression of NLRP3, ASC, and caspase-1 at the mRNA and protein levels and finally reducing IL- 1β and IL-18 secretion. Furthermore, TSPO was involved in the regulation of NLRP3 inflammasome as well as in treatment with TSPO ligands in TSPO knockdown microglial.

Microglial activation-induced neuroinflammation plays a vital role in the progression of neurodegenerative diseases, including AD (Sharma et al., 2020), PD (L’Episcopo et al., 2018), and perioperative neurocognitive disorders (PNDs; Saxena and Maze, 2018). In this study, BV2 microglial cells were activated by LPS, but the activation was significantly down-regulated with TSPO ligands. Therefore, we proposed a possible strategy to prevent or limit the occurrence and development of neurodegenerative diseases by using specific TSPO ligands. PK11195, as a ligand for TSPO, is used as a diagnostic tool for a variety of neuroinflammatory diseases (Harberts et al., 2013; Rissanen et al., 2014). Also, PK11195, which has neuroprotective potential (Milenkovic et al., 2015), suppresses microglial activation and has suppressive effects on neuroinflammation (Karlstetter et al., 2014). Midazolam is a benzodiazepine drug that acts on the brainstem and limbic system mainly through the benzodiazepine receptor (BZ receptor). It is widely used for clinical anesthesia (Prommer, 2020). Studies have found that midazolam can play a neuroprotective role by preventing lipid peroxidation and mitochondrial damage (Harman et al., 2012). Otherwise, TSPO drug ligands induce neuroactive steroid formation for the regulation of nervous system dysfunction (Porcu et al., 2016). In our study, we found that TSPO ligands PK11195 and midazolam inhibited the development of neuroinflammation through the NLRP3 inflammasome.

TSPO is known as the peripheral benzodiazepine receptor, which is mainly localized in the outer membrane of mitochondria in peripheral organs and the brain (Rupprecht et al., 2010). The widespread distribution of TSPO mitochondria indicates effects for this protein in regulating mitochondria-related function, including changes in mitochondrial capacity, ATP, and ROS production, leading to the evolution of neurodegeneration in patients (Betlazar et al., 2020). Meanwhile, changes in TSPO protein expression have major impacts on the regulation of inflammation (Setiawan et al., 2015). Mitochondria are regarded as a mediator of nucleate signaling through large molecular complexes, such as the NLRP3 inflammasome, and they activate inflammation through the release of mitochondrial danger-associated molecular patterns (Subramanian et al., 2013). ROS, as upstream signals of NLRP3 inflammasome activation, is required (Chen et al., 2017). The NLRP3 inflammasome affects the pathogenesis of the neurodegenerative disease (Sheedy et al., 2013). Activated NLRP3 inflammasomes can cleave pre-IL-1β and pre-IL-18 to generate mature IL-1β and IL-18. Inflammasomes are key players in the initiation and perpetuation of neuroinflammatory processes that require cytokine maturation, particularly IL-1β and IL-18 (Walsh et al., 2014). The NLRP3 inflammasome plays a vital role in the brain of patients with neurodegenerative disease (Heneka et al., 2018). There is increasing evidence that the importance of TSPO is based on its role in the process of NLRP3 inflammasome activation (Nakahira et al., 2011; Menu et al., 2012; Lee et al., 2016). Our current study shows that treatment of PK11195 and midazolam can inhibit the activation of BV-2 microglia and the production of ROS, thereby also inhibiting the expression of NLRP3, ASC and caspase-1 at the mRNA and protein levels and finally reducing IL- 1β and IL-18 secretion.

**Figure 4:**
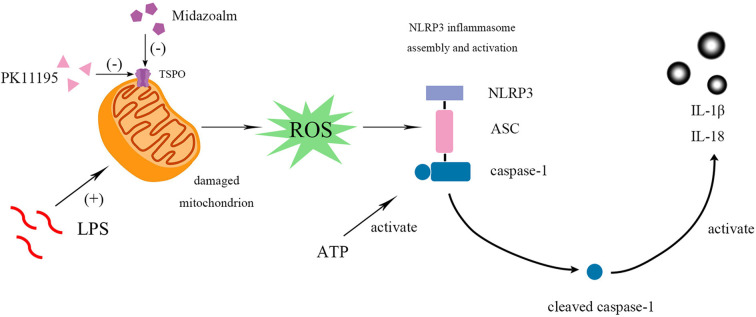
PK11195 or Midazolam down-regulated NLRP3 inflammasome and its downstream IL-1β and IL-18 in the reactivity of BV2 microglia cell. BV2 microglial cells were activated by LPS. LPS, priming stimuli, induce mitochondria to release ROS and then regulate NLRP3 inflammasome activation. ATP, an activation signal, triggers the specific activation of NLRP3. Finally, the cleaved-caspase-1 activates the IL-1β and IL-18. Both pretreatment and post-treatment with TSPO ligand can inhibit the activation of NLRP3 inflammasome and IL-1β and IL-18 expression. Collectively, these results indicate that TSPO ligands are promising targets to control microglial reactivity and neuroinflammatory diseases.

To further examine whether TSPO was involved in the regulation of NLRP3 inflammasome with TSPO ligands PK11195 or midazolam treatment, we established TSPO knockdown BV2 cells using siRNA transfection. Analysis of mRNA and protein expression revealed a significant reduction of TSPO in transduced cells compared with the control cells. We found that the levels of NLRP3 inflammasome were remarkably upregulated in TSPO knockdown compared with the control cells. Moreover, challenging the control cells with PK11195 or midazolam decreased the expression of the NLRP3 inflammasome, significantly different from the TSPO knockdown cells. However, the levels of NLRP3 inflammasome in the two groups were significantly different. The results indicated that TSPO was involved in the activation of NLRP3 inflammasome and in the modulation of inflammation with TSPO ligands treatment, but TSPO ligands may also interfere with mechanisms that are in part independent from TSPO (Bader et al., 2019; Figure 4). In the future, we will further explore the role of TSPO ligands in primary microglia cells and animal models, as well as other mechanisms independent of TPSO.

## Conclusion

The pretreatment or post-treatment of TSPO ligands inhibited the neuroinflammation caused by microglial activation through the NLRP3 inflammasome, interfered with the chronic inflammatory cascade, and disrupted the cytokine cycle, which may have a positive effect on the clinical treatment of neurodegenerative diseases. We might use TSPO as a target during in-depth clinical research and thus provide strategies for preventing and treating neurodegenerative diseases related to neuroinflammation.

## Data Availability Statement

The datasets presented in this study can be found in online repositories. The names of the repository/repositories and accession number(s) can be found in the article/Supplementary Material.

## Author Contributions

RZ designed the study. HF performed the experiments and wrote the manuscript with help from RZ, YLia, and YLiu. HF, YLiu, LS, NL, and BM analyzed the data. All authors contributed to the article and approved the submitted version.

## Conflict of Interest

The authors declare that the research was conducted in the absence of any commercial or financial relationships that could be construed as a potential conflict of interest.
