# Supplementary material for: TSPO Ligands PK11195 and Midazolam Reduce NLRP3 Inflammasome Activation and Proinflammatory Cytokine Release in BV-2 Cells
Source: Front Cell Neurosci. 2020 Dec 10;14:544431. doi: 10.3389/fncel.2020.544431 (PMC7759202; doi:10.3389/fncel.2020.544431)
Supplement: Supplementary file 1 [file Table_1.DOCX]

Supplement Figure 1. The effect of PK11195 alone or midazolam alone on NLRP3 inflammasomes and IBA-1 in BV-2 cells using western blot analysis. (A) NLRP3 expressions in BV-2 cell of each group. (B) ASC expressions in BV-2 cell of each group. (C) Caspase-1expressions in BV-2 cell of each group. (D) IBA-1expressions in BV-2 cell of each group.

Supplement Figure 2. Post-treatment of PK11195 or midazolam downregulated the activation of NLRP3 inflammasome and the release of IL-1β in the LPS+ATP-stimulated BV-2 cells. The cells were incubated with LPS (1 μg/ml) for 6 h, and then treated with PK11195 (0.5 μM) or midazolam (15 μM) for 2 h following ATP (1 mM) treatment for 2 h. (A-C) Comparison of NLRP3 (A), caspase-1 p10 (B), and IL-1β (C) expression in the BV-2 cells by group based on western blot analysis (n=5). (D) Comparison of IL-1β release in the medium of BV-2 cells by group using ELISA analysis (n=5). * *P* <0.05, ** *P* <0.01, compared with the control group; # *P* <0.05, ## *P* <0.01, compared with the LPS+ATP group.
