# Supplementary figures and images for: TSPO Ligands PK11195 and Midazolam Reduce NLRP3 Inflammasome Activation and Proinflammatory Cytokine Release in BV-2 Cells
Source: Front Cell Neurosci. 2020 Dec 10;14:544431. doi: 10.3389/fncel.2020.544431 (PMC7759202; doi:10.3389/fncel.2020.544431)

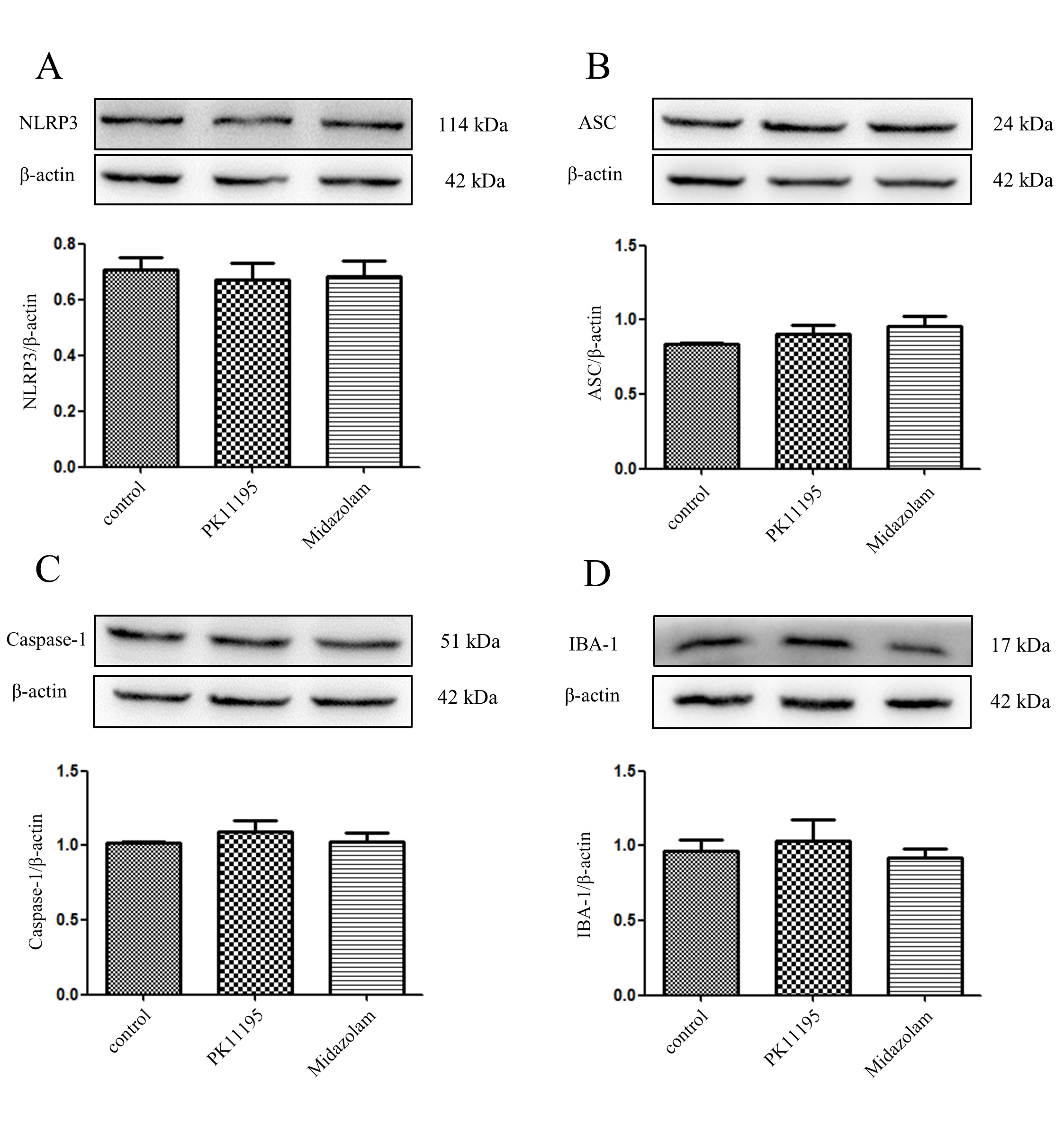

Supplement: Supplementary file 2 [file Image_1.JPEG]

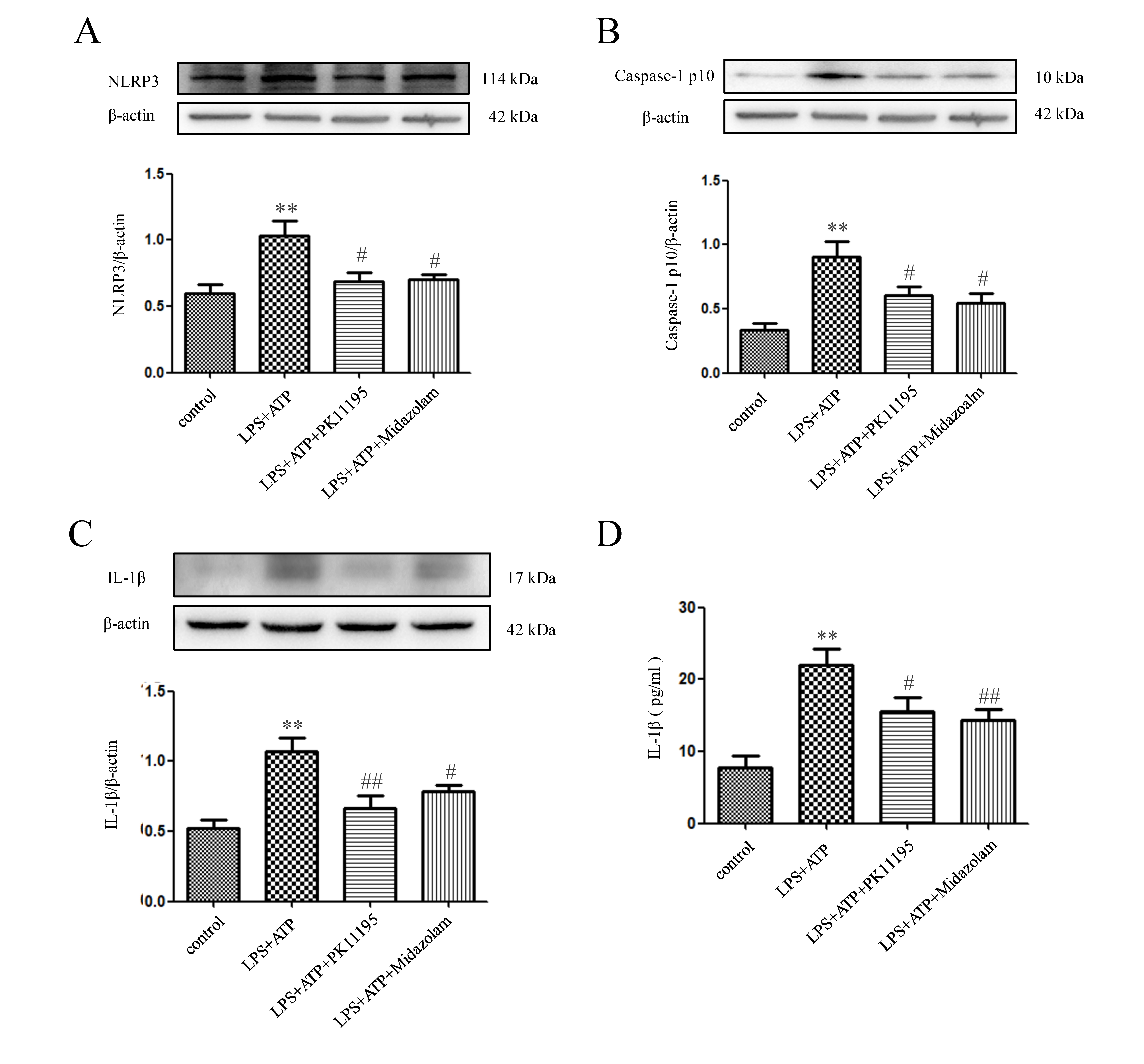

Supplement: Supplementary file 3 [file Image_2.JPEG]
